# Optimal design and setting of rotary strip-tiller blades to intensify dry season cropping in Asian wet clay soil conditions

**DOI:** 10.1016/j.still.2020.104854

**Published:** 2021-03

**Authors:** Md. A. Matin, Md. I. Hossain, Mahesh K. Gathala, Jagadish Timsina, Timothy J. Krupnik

**Affiliations:** a International Maize and Wheat Improvement Center (CIMMYT), Gulshan 2, Dhaka, 1212, Bangladesh; b Bangladesh Agricultural Research Institute (BARI), Joydebpur, Gazipur 1701, Bangladesh; c Bangladesh Wheat and Maize Research Institute (BWMRI), Nashipur, Dinajpur 5200, Bangladesh; d Institute for Study and Development Worldwide (IFSD), Sydney, 8/45 Henley Road, Homebush West, NSW 2134, Australia

**Keywords:** Conservation agriculture, Furrow backfill, Seedbed furrow, Soil tilth, Sustainable intensification

## Abstract

Fine-textured clayey soils dominate Asian rice fields that are kept either fallow or cultivated with non-rice crops after harvest of monsoon rice. Use of seeding machinery compatible with the principles of conservation agriculture on such soils, however, has not been promising. Under these conditions – which predominate the population and poverty dense areas of coastal South Asia – such machinery fails to open a furrow or throws excessive soil out of the tilled furrow during strip-till seeding. This results in a poor seed coverage at planting jeopardizing crop establishment. In response, this soil bin study investigated strip-tillage blade designs and settings to optimize rotary strip-till system for wet clay soil conditions common in South Asian rice fields. Three designs of C type rotary blade (conventional, medium and straight) and two blade settings (four and six blades per row; 50 and 100 mm cutting widths) were tested at three blade operating depths (50, 75, and 100 mm) using a tillage test rig and a soil bin, and a high-speed camera to understand the processes of soil cutting, throwing, backfilling, and creation of furrow seedbed. The soil bin soil consisted of a wet sandy-clay-loam soil with a moisture content of 28.2% (85% of field capacity) and was compacted to the bulk density of 1440 kg m^−3^. Using the test rig, rotary speed of the blades was maintained at 480 rpm and forward speed at 0.4 m s^−1^. At four blades per row setting, all blades created high amounts of optimum clods (1–20 mm size). The conventional and medium blades threw too much soil out of the strip-tilled furrow while the straight blade created adequate backfill at 75 and 100 mm operating depths. At 6 blades per row setting, all blades produced high amounts of backfill at any depths, but the straight blade also produced the highest amounts of optimum clods and a uniform furrow. Considering machine and energy costs, blade performance, and the necessity of minimizing soil disturbance in strip-tillage, our study indicates that the use of straight blades (four blades per row) operated at a depth of 75 or 100 mm are more ideal. These specifications are likely to enhance strip-tillage stand establishment in fine-textured soils with high moisture contents, though further work is needed under actual field conditions to confirm suitability of the proposed strip-till system for crop establishment in currently fallowed as well as the intensively cropped lands of Asia.

## Highlights

• Strip-tillage of Asian wet clay soil to intensify cropping resulted in poor seedbed.
• Conventional blades and setting (four per row) created optimum clods, but low backfill.
• Contrary, an improved blade (> 50 mm depth) created optimum clods and high backfill.
• Backfill increased further for 6 straight blades/row, but furrow volume increased.
• Straight blade (four per row, ≥ 75 mm depth setting) is recommended for Asian wet clay soil.

## Nomenclature

BD: blade design
CW: cutting width
dpi: dots per inch
*F_b_*: furrow backfill (kg m^−1^ or %)
kW: kilo Watt
OD: blade operating depth
rpm: revolution per minute
*V*: volume of the tilled furrow, m^3^
*W*: dry mass of soil remaining in the furrow, kg
*ρ*: dry basis bulk density of untilled soil (kg m^−3^)
°: degree

## 1 Introduction

Excess soil moisture is a commonly identified constraint in establishing a dry season, non-rice crop after puddled monsoon rice on the fine-textured clayey soils found on much of Asia’s rice lands. Cropping systems in these environments in South Asia often include two or more crops where monsoon (or ‘*kharif’*) season flooded rice is followed by winter (or ‘*rabi’*) season flooded rice or dryland crops such as maize, wheat, pulses or oilseeds, and in some cases also a third short-duration crop in spring (Islam et al., 2019; Timsina and Connor, 2001; Timsina et al., 2010). In rice based cropping systems, the most common practice for crop establishment in following dry season crops is tillage followed by manual broadcasting of seed. In many areas, however, fields are fallowed after the harvesting of monsoon rice. This is due in part to the clayey soil that retains excess soil moisture (Krupnik et al., 2015). Establishment of a second crop (dryland crop) on clayey and high-moisture content soils is challenging. Farmers typical practice of conventional tillage (full soil disturbance with 3–5 tillage passes using rotavators as reported in Mottalib et al., 2019) for land preparation is time and energy intensive. The time spent for land preparation can also cause delays in winter season sowing, precluding timely crop establishment and exposing winter or *rabi* crops to late-season terminal heat, drought, and soil salinity, in addition to the risk of waterlogging and lodging from early monsoon rains prior to crop harvest (Krupnik et al., 2015; Mondal et al., 2015). These challenges have increased interest in using two-wheeled tractor driven conservation agriculture (CA) seeders that can directly drill seeds (without any prior tillage) after rice is harvested and help reduce turnaround time considerably. According to Mottalib et al. (2019), CA seeders can reduce the turnaround time to zero in an optimum soil moisture condition. CA practices have been shown to save fuel, reduce labour, decrease crop establishment time, increase irrigation water use efficiency, and sequester organic carbon (0.1–0.5 t ha^−1^ year^−1^ or more) depending on the soil and cropping conditions, climate, and management practices (Baker et al., 2007; Kassam et al., 2018). However, CA requires specialized machinery (Erenstein and Laxmi, 2008; Fileccia, 2009; Sims et al., 2017) appropriate design of which depends on soil and cropping conditions (Celik and Raper, 2012; Munir et al., 2012; Vaitauskienė et al., 2015). Both Al-Kaisi and Yin (2005) and Gathala et al. (2020) suggested rotary strip-till seeders could aid in efforts to intensify dryland cropping by reducing land fallowing and assuring adequate yields while accruing environmental benefits including reduced greenhouse gas emissions and energy efficiency. In addition, strip-tillage has been shown to aid in improving soil organic carbon and soil biological activity, while buffering surface soil temperature and economizing soil moisture (Gathala et al., 2017; Haque et al., 2016; Morris et al., 2010).

**Fig. 1:**
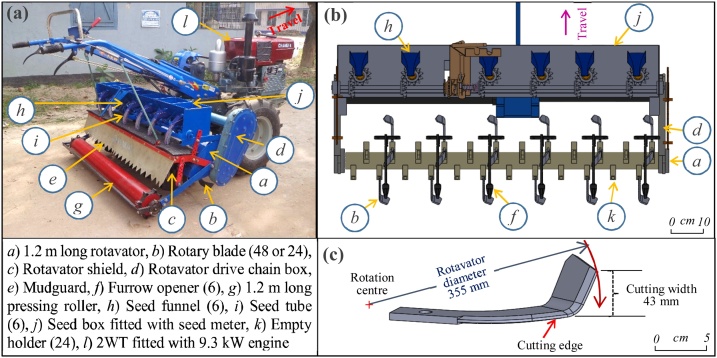
(a) A 9.3 kW two-wheeled tractor with an attached a six-row rotary seeder (1.2 m wide rotavator, fitted with 48 blades), (b) Modified strip-till configuration of the blades on the 1.2 m rotavator (every-other four alternate blades are removed, leaving four blades per row and six rows), and (c) the (right-hand) bent C blade (conventional blade) used with the rotary seeder.

Two-wheeled tractors (2WTs) fitted with rotary tillers are the main means of land preparation, seeding, and intercultural operations in many Asian countries. However, 2WTs have a low tractive ability that limits their use mostly in rotary tiller based agricultural operations. Sharda and Singh (2004) reported that the rotary tiller based machinery has negative draft requirement making them highly suitable for Asian wet clayey soils. Use of rotary tillers results in high soil pulverization and inversion and mixing of residues with soil (Asl and Singh, 2009; Gao et al., 2008). Two-wheeled tractor operated attachable 6-row rotary seeders (known as power tiller operated seeder or PTOS with bent C rotary blades) are increasingly common among smallholder farmers in South Asia (Fig. 1a). Due to high density of the rotary blades (48 blades on a 1.2 m rotor) and rotor’s high-speed (450–500 rpm) compared to common rotary tillers (18 blades on a 0.6 m rotor, 220–250 rpm), these seeders allow land preparation and seeding in a single-pass (Haque et al., 2010; Hossain et al., 2009; Krupnik et al., 2013). These rotary seeders are commonly converted into ‘strip-till seeders’ by removing every alternating four blades (totaling 24 blades for six rows) to eliminate inter-row soil tillage (Hossain et al., 2009; Matin et al., 2015). During operation, strip-till seeders produce narrow furrow strips, often 50–60 mm wide and 50 mm deep (Dobbratz et al., 2019; Reeder, 2002), while simultaneously sowing and covering seeds, and leaving the inter-row undisturbed.

However, CA is knowledge intensive: strip-tillage machinery is more complicated than conventional tillage implements, consisting of different soil engaging tools (Lei, 2019). Commonly used 2WT operated small scale strip-till seeders in Bangladesh, for example, consist of a rotavator with mounting of blades (for soil tilling and pulverizing), a rotavator mudguard (for capturing thrown clods and assist in pulverization), a seed metering system, furrow openers (for placing seeds into the furrows) and a levelling roller (for leveling and compacting soil surface after seeding) (Fig. 1a). Although, all above components of the strip-till seeders are important in their respective role and functions, evidence indicates the most important soil engaging component is the machine’s rotavator (rotary blades). Specifically, appropriate blade designs are needed to produce identical furrow parameters and improve seed to soil contact and placement for optimal crop establishment under strip-tillage (Matin et al., 2014; Yang et al., 2018).

Commercially available C blades (Fig. 1b and c) that come with most commercially available rotary seeders are designed for full soil tillage. Even if portions of the blades (bent sections) are removed to permit strip-tillage, use of these blades tends to result in excessive soil throw out of the furrow strips mainly during the exit of the blade from the soil (Asl, 2006; Hossain et al., 2009; Matin et al., 2014). This results in inadequate furrow backfill and poor seed coverage (Hossain et al., 2009; Lee et al., 2003; Matin et al., 2014), increased seed predation, reduced emergence, and suboptimal plant stands. To reduce soil throw during rotary strip-tillage, blades of different shapes have been designed and tested. Beeny and Khoo (1970) reported that blade shape has a great effect on the specific power requirement, with the bent C blades requiring considerable power to cut, throw and pulverize the soil. The rotary power requirement for conventional tillage was 19–29% less for bent C blades than L blades (Asl and Singh, 2007a, 2007b; Sharda and Singh, 2004). Hassan (1980) alternatively reported that wedge-shaped blades minimize soil cutting energy requirements for tillage. In contrast, straight C blades can produce high quality seedbed furrow while minimizing soil throw and increasing furrow backfill (Matin et al., 2014). To optimize performance, Hongbo et al. (2020) examined three types of straight blade profiles (Archimedean spiral, logarithmic spiral, and sinusoidal-exponential spiral) with four edge-curve angles (30°, 40°, 50°, and 60°) using Discrete Element Method simulation for strip-tillage to show that the Archimedean spiral blade threw the highest number of soil particles and required the greatest torque. They, therefore, recommended a larger edge-curve angle of the straight blade when low soil disturbance and low torque requirements are desired. Yang et al. (2018) compared straight C, bent C, and hoe blades for rotary strip-tillage in a clay loam paddy soil, although they found that none of the blades were suitable for rotary strip-tillage in a clay loams. They suggested a mixed blade setting for each row (two hoe blades at the center and two straight blades on the sides) for strip-tillage under these conditions.

The energy requirement for strip-till soil cutting and throwing, and pulverization also depends on rotary speed, operating depth, and cutting width of the blades (Asl and Singh, 2007a; Celik et al., 2008; Hendrick and Gill, 1971; Kosutic et al., 1997; Matin et al., 2015). Higher rotary speed can pulverize the soil and achieve high soil tilth, but it can also reduce the furrow backfill (Matin et al., 2014, 2015; Yang et al., 2018). The specific power and energy requirements for strip-tillage also depend on blade width and blade numbers per flange that cut a single furrow, with requirements increasing with blade width and number (Ahmad, 1986; Asl and Singh, 2007a, 2007b). Under strip-tillage, evidence suggests that the number of blades per flange should be minimized and peripheral distance between blades maximized to allow residue flow with no clogging of rotors or furrow openers (Gill and Berg, 1968).

Tillage depth similarly has a direct effect on soil pulverization and power requirement (Celik et al., 2008; Hendrick and Gill, 1971). The movement of tilled soil depends on the ratio of blade operating depth and rotor radius (Salokhe and Ramalingam, 2002). Kulaya and Singh (2019) reported that the mean mass diameter of clods in case of J blades gradually increased with the blade operating depth in a medium textured soil. FAO (Food and Agriculture Organization of the United Nations) (2000) suggested minimizing tillage down to the seeding depth only, as tilling deeper is costly and could accelerate soil moisture loss. However, in some conditions, tilling deeper than the seeding depth may be necessary to break hardpans, apply fertilizer with seed, and reduce the deleterious effects of root diseases (e.g. *Rhizoctonia solani*) on developing seedlings (Gill et al., 2001; Zeng et al., 2017).

Blade cutting width is another parameter that has a direct effect on soil pulverization and power requirement. Ahmad (1986) found that the power requirement for rotary strip-tillage in a soil bin increased with the cutting width of L blades due to greater soil cutting force. However, the specific energy requirement was reduced with an increase of blade width. Ahmad (1986) also reported that the increase of rotary and forward speeds at a constant bite length improved soil tilth for the wide L blades but with no significant change for narrow L blades. Matin et al. (2014, 2015) showed that an 8 mm wide straight blade produced similar soil tilth as a 43 mm wide blade while using 25% less power under strip-tillage. Zhao et al. (2018) similarly preferred straight C blades over conventional C blades for strip-tillage due to lower torque requirement, soil resistance, soil-residue mixture, and soil throw resulting in a neat seedbed. Therefore, a reduction of the blade cutting width has been suggested (e.g. Ahmad, 1986; Ju, 2007; Matin et al., 2014, 2015) as a method to minimize the soil acceleration force, soil throw, and energy requirement.

The quality of soil tilth is important for seed-soil contact and seed emergence and rotary blades may or may not have effects on formation and distribution of soil particles and clods (Chertkiattipol et al., 2008; Salokhe and Ramalingam, 2001, 2002). Matin et al. (2014, 2015, 2016) however suggested that straight blades with inside chamfered cutting edges improved furrow backfill, produced ideal soil tilth for seed germination, and reduced torque and energy requirements for strip-tillage. However, preliminary field tests conducted by CIMMYT (2017) on moist clay soils in southern Bangladesh showed that neither the conventional C blades nor the straight blades suggested by Matin et al. (2014, 2016) could create suitable strip-tilled furrows at the common blade setting of four blades per row and 50–60 mm blade operating (tillage) depth. An improved understanding of soil cutting, throwing, and backfilling processes of various blade designs at a range of operating depths and cutting widths is therefore necessary for optimal design of strip-till seeders to achieve *rabi* season crop establishment with strip-tillage on South Asia’s heavy (fine textured) and often high-moisture containing soils. Since the soil carrying ability of a certain width of blade is fixed (Celik and Altikat, 2008), an increase in either the operating depth or the cutting width of blades (i.e., furrow cutting width) is likely to increase soil pulverization and amount of soil thrown back into the furrow, potentially providing improved seed and furrow cover. In response, we undertook a soil bin study to optimize blade design and setting to facilitate identical seed furrow parameters using strip-till seeding on a moist clay soil, while also identifying appropriate blade design and settings for 2WT attached strip-till seeders for fine textured soils. Soil bins were chosen as they have been used extensively to evaluate rotary blades at various forward and rotor speeds and operating depths and cutting widths to understand the soil cutting, pulverizing, throwing, and backfilling processes and develop understanding of soil-tool interaction without influence of exogenous factors across soil types and climates (Ahmad, 1986; Ani et al., 2018; Asl and Singh, 2007a, 2007b; Asl and Singh, 2009; Chertkiattipol and Niyampa, 2010; Lee et al., 2003; Matin et al., 2014, 2015, 2016; Tagar et al., 2015).

## 2 Materials and methods

### 2.1 Experimental procedure

#### 2.1.1 Soil bin system and soil collection and preparation

**Fig. 2:**
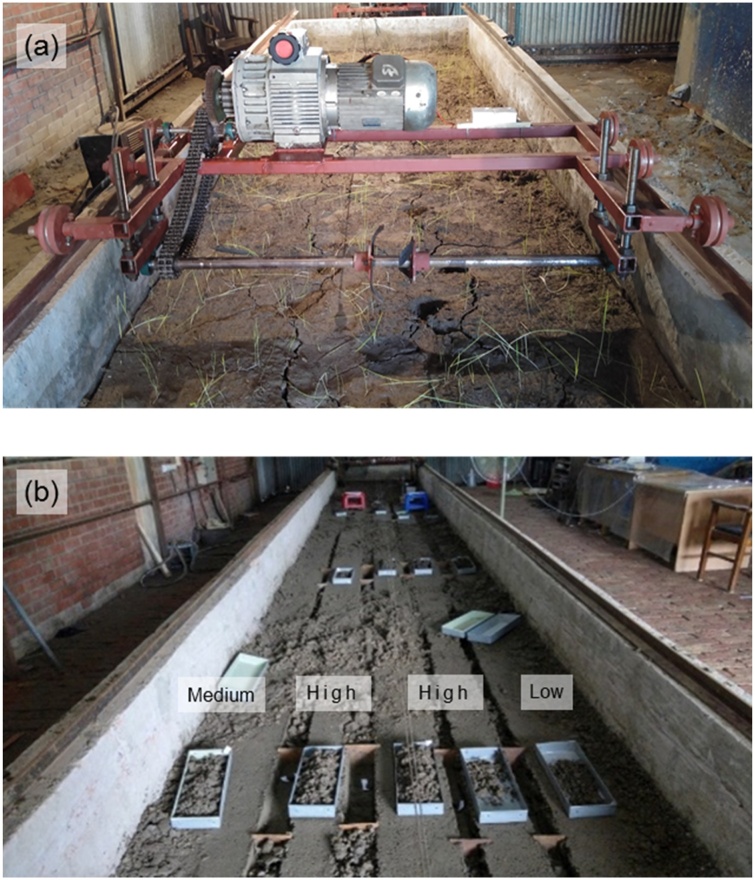
(a) Soil bin apparatus and rotary axle shown with blade holders prior to experimentation, and (b) depiction of furrow backfill, soil tilth and other furrow data collection in the soil bin after test runs.

**Fig. 3:**
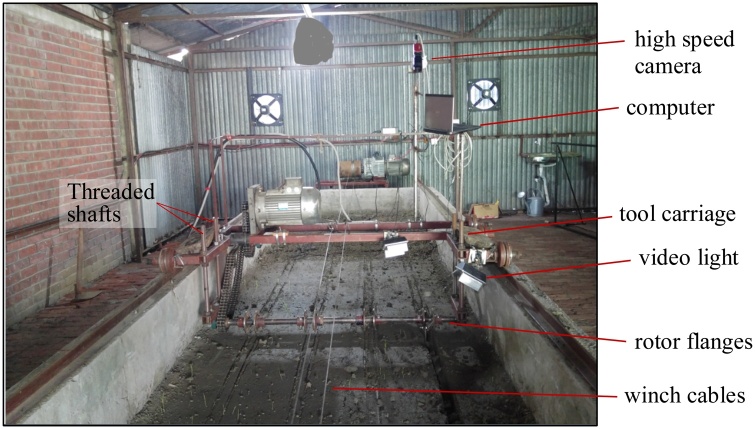
Tillage test rig at the Farm Machinery and Postharvest Process Engineering Division at the Bangladesh Agricultural Research Institute, Gazipur, Bangladesh.

This study was conducted using an indoor soil bin system located at the Bangladesh Agricultural Research Institute, Gazipur, Bangladesh. The soil bin was 14 m long, 1.7 m wide and 0.5 m deep, and was equipped with a seeding test rig (Fig. 2, Fig. 3) designed to maintain accurate blade operating depth and forward and rotary speeds, while operating a set of four or six blades to cut a strip-tilled furrow.

The soil bin was filled with sandy-clay-loam soil (47.2% sand, 22.0% silt and 30.8% clay) with field capacity (FC) soil moisture content (SMC) of 33.4 ± 0.4%. The soil was collected from a field (top 150 mm soil layer) in south-western Bangladesh analogous to many sandy-clay-loams used to cultivate monsoon season rice that remain excessively wet in the early dry season after rice is harvested (Mondal et al., 2015). Our study focused on quantifying the effect of blade design and settings on strip-tilling performance in clayey soil; as such, any foreign material present in the soil would interfere the true effect of blade design and setting. Therefore, the soil was manually pulverized, and grasses, plant residues and other foreign materials were removed from the soil in a fashion similar to other studies studying soil-tool interaction for strip- or conventional tillage (Ahmad, 1986; Asl and Singh, 2007a, 2007b, 2009; Kulaya and Singh, 2019; Lee et al., 2003; Tagar et al., 2015). Cleaned soil was filled into the bin to provide 125 mm deep soil bed ready for the test. To obtain consistently moist soil beds throughout the experimental runs, we added a pre-determined amount of water to reach a targeted SMC of 28.2 ± 0.6 (84.5% of FC). At any higher soil moisture content, the soil became too wet to be tilled or pulverized to permit furrow formation. We conversely did not study lower soil moisture contents as waiting for soils to dry before tillage would mean additional delays for farmers in timely crop establishment, which would result in either missing of the window of opportunity for growing a second crop or delayed establishment leading to increased abiotic risks to achieving adequate yields (Rahman et al., 2015).

One day after adding the predetermined amount of water, the soil was loosened, mixed, levelled to a set height, and compacted using 50 passes of a roller (240 kg m^−1^) to obtain a compacted soil bed of 1440 kg m^−3^ bulk density resembling the field condition. The soil bed was divided into three sections, each section of 4.0 m long, to accommodate four strip-till test runs (i.e., four different strip-tilled furrows) across the width (Fig. 2b). Thus, twelve treatment runs (each run produced one 4.0 m long furrow strip) were completed in each batch using the whole bin.

#### 2.1.2 Experimental treatments

A three-factor factorial experiment was conducted in a randomized complete block design with three replications. The three factors were blade design, blade operating depth, and cutting width with 3-, 3- and 2-levels per factor, respectively. The treatments included: Factor A (blade design) with three treatments (conventional or bent C, medium or half-width bent C, and straight blades), Factor B (blade operating depth) with three treatments (50 mm, 75 mm, and 100 mm), and Factor C (cutting width) with two treatments (50 mm using 4 blades per row and 100 mm using 6 blades per row).

**Fig. 4:**
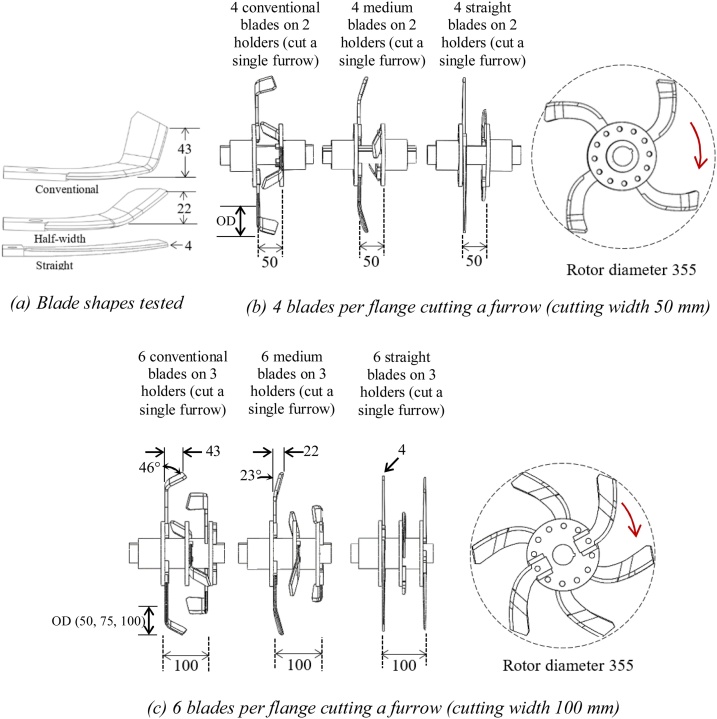
(a) Blade shapes tested, (b) 4 blades per flange set to cut a 50 mm wide furrow (2 blade holders spaced at 50 mm, each holding 2 blades), and (c) 6 blades per flange set to cut a 100 mm wide furrow (3 blade holders spaced at 50 mm, each holding 2 blades). Blade operating depth (OD) varied at 50, 75 and 100 mm. Dimensions are in mm unless stated otherwise.

The conventional blade used the left- and right-hand bent C blades with a cutting width of 43 mm and angle 46°, which is commonly used with the commercially available rotary seeders for seeding with land preparation (as marked in Fig. 1b and c). The medium blades were made by modifying the conventional blade and had a cutting width of 23 mm with tip angle 23° while the straight blades were made of mild steel plates and had a 4 mm cutting edge thickness with tip angle 0° (Fig. 4a). All treatments used a rotor diameter of 355 mm. During test runs, any previously tilled furrows were covered by polyethylene sheets to avoid contamination of the tilled furrow soil due to the soil thrown from the subsequent test runs.

#### 2.1.3 The rotary tiller test rig and operation of blades

The rotary tiller was fitted with four or six blades per row using two or three sets of blade holders (spaced at 50 mm, each holding two blades with 180 angular spacing in between), respectively as shown in Fig. 4b and c. The blade holders were fitted out of phase by 90° or 60° so that one of the blades hit soil at every 90° or 60° turn of the rotor, respectively (Fig. 4b and c). No rotavator shield or cover, which typically are used to protect the machine operator from flying soil debris, was used. This also avoided the interaction of thrown soil clods with a cover that would have altered the true effect of soil-blade interaction. Therefore, the clods were free to move in any direction as governed by the blade shape and setting effect on throwing. The rotor was operated in a forward rotation as recommended for strip-tillage as reverse rotation throws tilled soil out of the furrow strips potentially reducing soil cover over seeds (Asl and Singh, 2007b, 2009; Lee et al., 2003). The forward travel and rotary speeds of the rotor were maintained constant at 0.4 m s^−1^ (using a 3-phase, 5.5 kW, 1400 rpm geared electric motor) and 480 rpm (using a 3-phase, 7.5 kW, 1400 rpm geared electric motor and chain-sprockets), respectively resembling farmers’ practice.

#### 2.1.4 High-speed video camera to understand soil cutting and backfilling processes

During operation of the rotary blades at high-speed (480 rpm), blades can cut, pulverize, and throw soil. The patterns of soil cutting and throwing vary as per the blade design and its operation (Matin et al., 2014, 2016), and such patterns are not possible to observe with the naked eyes. Hence, following Matin et al. (2014, 2016) and Lee et al. (2003), a high-speed video camera was used to capture imagery allowing visualization of these patterns. The video camera (Promon 501, AOS Technologies AG, Baden-Daettwil, Switzerland), was fitted on the test rig to capture the images (565 frames s^−1^, 320 × 642 dpi) of the process of soil cutting, pulverizing, throwing, and backfilling and understand actions that help achieve a good quality furrow seedbed and help explain the results. Images were acquired on a laptop as RAW3 files using Promon Studio software (v3.8.3.1, AOS Technologies AG, Baden-Daettwil, Switzerland) during the test runs. The images were analyzed with Promon Studio software.

### 2.2 Data collection and processing

After each day of testing, the soil bin was left uncovered overnight for drying and hardening of the clods and the furrow walls before sampling for data collection in the following day. The data collection process for each of the twelve strip-tilled furrows included cleaning away the soil clods thrown outside the furrow edges (and thus became potentially unavailable to cover seeds), collecting loose soil (to calculate furrow backfill) in trays from inside a 500 mm furrow section and oven drying it at 105 °C for 24 h, weighing and hand sieving the soil through 20 mm and 1 mm sieves. These sieve (or clod) sizes were selected based on Berntsen and Berre (1993); Lee et al. (2003) and Matin et al. (2014, 2016), who targeted achieving 1–20 mm clods in the seedbed for optimum seed-soil contact.

#### 2.2.1 Furrow width and depth

The mean vertical distance between the untilled soil surface and the cut furrow bottom at the center of the furrow cross-section was taken as the average furrow depth. The width of the furrow was taken as the mean horizontal distance between the two edges of the furrow measured at the soil surface perpendicular to the travel direction. The furrow center depth and top width were calculated as means of readings taken at 10 consecutive locations spaced at 50 mm along the furrow length using a 0.5 mm graduated ruler, as described by Matin et al. (2014, 2016).

#### 2.2.2 Furrow backfill, furrow volume, and furrow shape

Furrow backfill was expressed as the amount (kg m^−1^) or % of the untilled soil retained in the furrow after strip-tillage and was calculated as the dry weight of the tilled soil collected from inside the 500 mm furrow section following Matin et al. (2014). A high backfill is desired to adequately cover seeds to ensure seed emergence and reduce the risk of bird damage. The furrow backfill (%) was calculated as:

(1) *F_b_ = 100 W/Vρ*

here, *F_b_* is furrow backfill, *W* is dry mass of soil remaining in the furrow section (kg), *V* is the volume of tilled furrow section (m^3^), and *ρ* is bulk density (dry basis; kg m^−3^) of the untilled soil. The volume of soil disturbance or furrow volume (cm^3^ m^-1^) was measured using the sand replacement method (Matin et al., 2014). Furrow shape was also measured following Matin et al. (2014). A soft ductile wire was carefully molded to the furrow shape and then transcribed onto graph paper to obtain the furrow shape.

#### 2.2.3 Soil tilth

Backfilled soil clods and particles collected from the furrow section were divided into three groups after oven drying based on their sizes: fine particles (<1 mm size), optimum clods (1–20 mm size) and large clods (>20 mm size). Soil clods and particles were expressed in both amount (kg m^−1^) and percentage (%). The optimum clods were calculated as the weight or the percentage of the dried soil that passed through a 20 mm sieve but retained on a 1 mm sieve. Fine particles were defined as those that passed through 1 mm sieve.

### 2.3 Data analysis

Data on width, depth, shape and volume of the furrow, furrow backfill (in amount and percentage), and tilth quality parameters such as fine particles, and optimum and large clods (in amount and percentage) collected for each treatment were subjected to statistical analysis by factorial analysis of variance (ANOVA) using SAS (V9.4 statistical software; Littell et al., 2006). Where significant differences were found, treatment means were separated by using Tukey’s honestly significant different *post-hoc* test (HSD test).

## 3 Results and discussion

The ANOVA of the seedbed soil and furrow parameters showed significant effect (*P* = 0.01) of blade design, blade operating depth, and cutting width on all parameters except optimum clods, fine, and large particles considering blade design and operating depth (Table 1). The interaction effect of blade design and operating depth was significant (*P* = 0.01) for furrow depth (*P* = 0.006), amount of furrow backfill (*P* = 0.007), and optimum clods (*P* = 0.023). The interaction of blade design and cutting width was significant only for furrow depth (*P* = 0.018) and percent furrow backfill (*P* = 0.041). Considering the effect of operating depth and cutting width, interactions were significant (*P* = 0.001) for furrow volume, amount of furrow backfill, and for large clods. Three-way interaction effect was significant (*P* = 0.01) for furrow depth only (Table 1).

**Table 1:** Individual effects of blade design, operating depth, and cutting width on various strip-till furrow quality parameters.

Source	Furrow width (mm)	Furrow depth (mm)	Furrow backfill (kg m^−1^)	Furrow backfill (%)	Optimum clods (kg m^−1^)	Optimum clods (%)	Furrow volume (cm^3^ m^−1^)	Fine particles (kg m^−1^)	Large clods (kg m^−1^)	Fine particles (%)	Large clods (%)
*Blade design (means across operating depth and cutting width)*											
Conventional	97.2a	82.8a	2.28b	24.3b	1.40b	64.6	5851a	0.02b	0.85b	1.0	34.4
Medium	87.1b	76.6b	2.12b	27.6b	1.30b	64.6	4759b	0.02b	0.80b	1.3	34.1
Straight	90.2b	80.2a	4.03a	47.9a	2.77a	71.4	5658a	0.04a	1.21a	1.2	27.4
*Operating depth (means across blade designs and cutting width)*											
50 mm	84.8b	58.8c	1.93c	32.7	1.27b	68.5	3760c	0.02b	0.64b	1.3	30.2
75 mm	93.1a	75.1b	2.53b	32.1	1.66b	66.0	5141b	0.03b	0.84b	1.1	32.9
100 mm	96.6a	105.7a	3.97a	34.9	2.55a	66.1	7367a	0.04a	1.38a	1.1	32.8
*Cutting width (means across blade designs and operating depth)*											
50 mm	68.05b	78.7b	1.40b	26.2b	1.03b	72.7a	3624b	0.02b	0.35b	1.4a	25.9b
100 mm	114.93a	81.0a	4.22a	40.3a	2.62a	61.0b	7222a	0.04a	1.56a	0.9b	38.0a
*Analysis of variance (P-values)*											
Blade design (BD)	<0.0001	<0.0001	<0.0001	<0.0001	<0.0001	0.0919	<0.0001	<0.0001	0.0002	0.2371	0.086
Operating depth (OD)	<0.0001	<0.0001	<0.0001	0.3792	<0.0001	0.7196	<0.0001	<0.0001	<0.0001	0.481	0.6825
Cutting width (CW)	<0.0001	0.0227	<0.0001	<0.0001	<0.0001	0.0002	<0.0001	<0.0001	<0.0001	0.0023	0.0001
BD × OD	0.3575	0.0062	0.0075	0.621	0.0227	0.5644	0.7983	0.1551	0.4118	0.3024	0.5196
BD × CW	0.0997	0.0184	0.057	0.0411	0.217	0.5979	0.1532	0.8798	0.261	0.4647	0.6359
OD × CW	0.5672	0.4731	<0.0001	0.8807	0.0538	0.1353	<0.0001	0.4302	<0.0001	0.3376	0.1168
BD × OD × CW	0.218	<0.0001	0.4704	0.483	0.6703	0.8481	0.1827	0.1381	0.6634	0.3499	0.8518

Means within each series followed by the same letter do not differ significantly according to the Tukey’s honestly significant difference post-hoc test (HSD test).

### 3.1 Furrow width, depth, and volume

Irrespective of the blade setting (i.e., four or six blades per row), all the blades cut wider furrow than the cutting width of the blades as the blades shattered the furrow walls during cutting, mainly during exit of the blade out of the furrow as observed in the high-speed image analysis. A similar phenomenon of rotary strip-till furrow cutting has been reported in literature (e.g., Matin et al. (2014). With the longest (43 mm) sidelong section, the conventional C blade broke wider (> 43 mm) chunks of soil with each bite. Thus, the furrows made by these blades were significantly (*P* = 0.01) wider by 10.1 mm and 7.0 mm than the medium and straight blade, respectively. The shallowest blade operating depth (50 mm) created an 11% narrower furrow width than the other two operating depths of 75 and 100 mm. Furrow width increased significantly (by about 68%) with increase of blade cutting width and number from four (50 mm width) to six (100 mm width) (Table 1). This suggests that to obtain a 50 mm wide furrow (common for 2WT operated CA seeders aiming to sow seeds with minimum soil disturbance), the cutting width of blades needs to be set approximately at 40–45 mm, consistent with other studies on a sandy loam soil (Matin et al., 2014, 2016). This reduction of cutting width setting could also potentially help reduce power requirement (Ahmad, 1986: Matin et al., 2015) and operating cost, though verifications under controlled field trial conditions and with crop residue retention are needed.

**Fig. 5:**
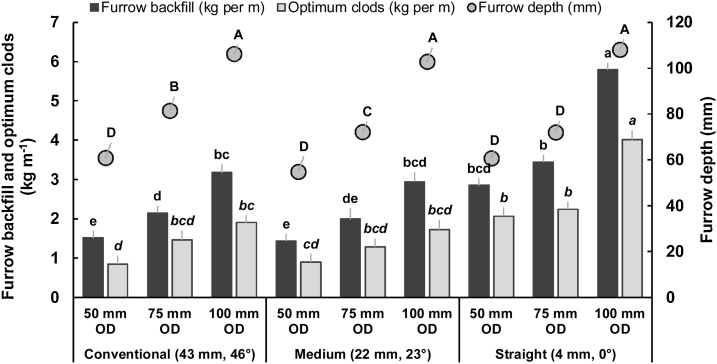
Interaction effects of blade design × blade operating depth on furrow depth, furrow backfill and optimum clods. Means within each series followed by the same letter do not differ significantly (p = 0.05). OD = blade operating depth.

**Fig. 6:**
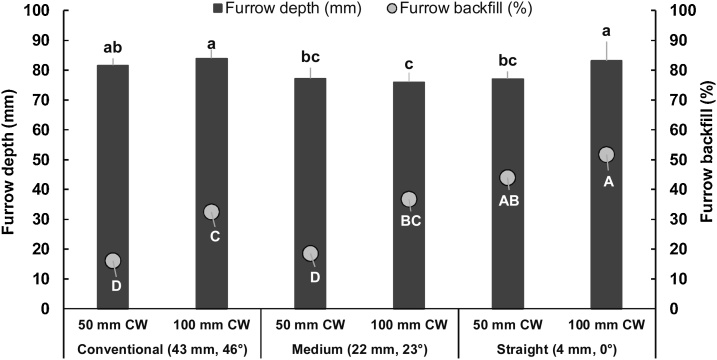
Interaction effects of blade design × operating width on furrow depth and furrow backfill (%). Means within each series followed by the same letter do not differ significantly (p = 0.05). CW = cutting width.

Furrow depth significantly (*P* = 0.01) increased with the blade operating depth, irrespective of blade design (Fig. 5). All the blades tilled furrows slightly deeper than the blade operating depth due to shattering of bottom soil. The exception, however, was the straight blade at 75 mm operating depth, which left uncut bottom ridge along the furrow center which would potentially result in deflection and shallow placement of seeds, and hence would be less suitable for seeding. Along with the blade designs at 50 mm and 100 mm operating depths, furrow depth did not vary significantly, but these blade designs produced variable furrow depths at 75 mm operating depth with following the order of deepest to shallowest as conventional > medium > straight (Fig. 5). The wider cutting width of 100 mm produced a 2.3 mm deeper furrow than the narrower cutting width (50 mm) for the same blade operating depth (Table 1). An interaction effect of blade design and cutting width was significant (*P* = 0.02), producing a shallower furrow depth with medium and straight blade at 100 mm and 50 cutting width, respectively than conventional and straight blades at 100 mm cutting width (Fig. 6). Hence, the straight blades appear to be as efficient as the conventional blades in cutting furrows and maintaining optimum furrow depth in a moist sandy-clay-loam soil.

The furrow volume cut by the blades was significantly greater (*P* = 0.001) for the conventional and straight blades compared to the medium blade as observed in other studies (e.g., Matin et al., 2014). The increase was largely linear, with the increase of both the operating depth and cutting width. It therefore followed the same trend as the furrow width and depth. There were two significant (*P* = 0.01 and 0.02) interactions (blade design by operating depth and blade design by cutting width) showing positive effects on the furrow depth (Fig. 5, Fig. 6).

### 3.2 Furrow backfill

Both the amount and percentage of furrow backfill were significantly influenced by blade design and cutting width. But operating depth significantly affected the amount of backfill only, indicating that the amount of backfill would probably be a more meaningful parameter than the percentage of backfill when the practical availability of tilled soil to cover seeds is being assessed. Irrespective of the blade design and operating depth, the straight blade and the 100 mm operating depth produced the highest amount of backfill (4.03 and 3.97 kg m^−1^, respectively). The straight blade produced twice the amount of backfill than by the other two blade designs, whereas the 100 mm operating depth produced 56% and 105% higher amount of backfill in furrows than the 75 mm and 50 mm operating depths, respectively (Table 1). The interaction effects showed that the straight blade with 100 mm operating depth produced the highest (*P* = 0.01; 5.8 kg m^−1^) amount of backfill while the conventional and medium blades at 50 mm operating depth produced the lowest amount (1.7 kg m^−1^) (Fig. 5). On the other hand, 100 mm operating depth at 100 mm cutting width produced the highest amount of furrow backfill followed by 75 mm operating depth at 100 mm cutting width. The straight blade achieved 83% greater furrow backfill than the conventional and medium blades; this was 53% greater with the 100 mm than the 50 mm cutting width. The straight blade at 100 mm cutting width had the highest backfill (51.7%; *P* = 0.06) while conventional and medium blades at 50 mm cutting width had the lowest (16.1–18.6%) (Fig. 6).

Visual assessment suggested that for the four blade per row setting (50 mm cutting width), the backfill produced by the straight blade at 75 or100 mm blade operating depth was adequate to cover the maize or wheat seeds and maintain optimum soil-seed contact, but that by the conventional or medium blade was likely to be insufficient. When the number of blades per row was increased to six (100 mm cutting width), all the blades produced sufficient amounts of backfill to cover seeds at all blade operating depths. Similarly, the straight blade produced the highest amount of furrow backfill with the six blades per row setting, showing its potential superiority over conventional or medium blade for strip-tillage, confirming Matin et al. (2014) and Yang et al. (2018).

### 3.3 Furrow tilth

There was a significant effect (*P* = 0.01) of blade design, blade operating depth, and cutting width on furrow tilth quality (defined by the amounts of desired optimum clods, and unwanted fine particles and large clods). The distribution pattern of both fine particles and large clods followed the same trend. The particle distribution pattern was more distinct with straight blade design, 100 mm operating depth, and 100 mm cutting width over others. All the blade types, irrespective of blade operating depth and cutting width, produced negligible amounts (0.02–0.04 kg m^−1^; 0.9–1.4%) of fine particles or dusts (Table 1). Thus, it is less likely that a surface crust would occur upon irrigating the tilled soil of the field from where soil was collected and used in the current soil bin experiment, though this hypothesis requires validation under field conditions.

**Fig. 7:**
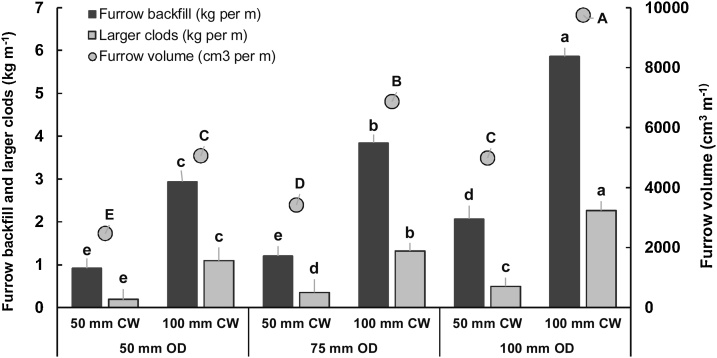
Interaction effects of blade operating depth × cutting width on furrow backfill, furrow volume and amount of large clods. Means within each series followed by the same letter do not differ significantly (p = 0.05). OD = operating depth; CW = cutting width.

The rotary and forward speeds used in this study provided a short bite length of 13 mm. Small soil slice cutting was observed per bite. This helped achieve high percentage (61.0–72.7%) of optimum clods across treatments. Among the blades tested, the straight blade produced a significantly *(P=*0.01) higher amount of optimum clods (2.77 kg m^−1^) compared to conventional or medium blade (1.35 kg m^−1^), though the percent of optimum clods did not differ significantly across blade designs. The higher percentage of optimum clods was maintained in the straight blade at 100 mm operating depth (Fig. 5). The amount of large clods observed was significantly (*P* = 0.01) greater for the straight blade (1.21 kg m^−1^) compared to other two blades (0.82 kg m^−1^); it was also significantly greater (*P* = 0.01) for 100 mm operating depth and 100 mm cutting width compared to other operating depths and cutting width (Fig. 7). Interactions revealed the greatest (*P* = 0.02) amount of optimum clods (4.0 kg m^−1^) for the straight blade with 100 mm operating depth, but similar amounts (1.29–2.25 kg m^−1^) for straight blades with 50 or 75 mm operating depths, and for conventional and medium blades with 75 or 100 mm operating depths (Fig. 6). The 100 mm operating depth with 100 mm cutting width had the greatest amount, while 50 mm operating depth with 50 mm cutting width had the lowest amount, of large clods, respectively (Fig. 7).

### 3.4 Furrow shape

All three furrow parameters (furrow backfill, optimum clods size, and furrow volume) for good crop establishment were associated with the straight blade design and 100 mm operating depth for both cutting width combinations (Table 2). The furrow shapes produced by the blades varied widely depending on the blade shape, operating depth, and blade setting. The conventional and straight blades tended to produce U-shaped furrows with loose soil at the furrow bottom indicative of improved conditions for seed coverage, while the medium blade tended to produce V-shaped furrows with a narrow bottom, limiting potential seed coverage (Table 2). The straight blade produced the most uniform furrow by taking the closest dimension of the targeted furrow section (operating depth of 50, 75 or 100 mm and cutting width of 50 or 100 mm). Furrow uniformity increased with all the blades when operated deeper (producing a regular shape furrow with the furrow top edge little wider than the bottom). The furrow top edge was distorted during exit of the blade out of the furrow and thus the furrow top became little wider than the furrow bottom. The medium blade, due to its narrow-bent section (23 mm wide), tended to leave an uncut ridge at the middle of the furrow resulting in shallow furrow depths (Table 2).

**Table 2:** Effects of blade design and blade setting (cutting width) on the furrow shape and furrow quality parameters.

	Conventional blade	Medium blade	Straight blade	Conventional blade	Medium blade	Straight blade
	Cutting width 50 mm (4 blades per row)			Cutting width 100 mm (6 blades per row)		
*Blade operating depth 50 mm*						
	b^1^ = 1.045 (21%) o^2^ = 0.81 (78%) v^3^ = 3420	b = 0.369 (14%) o = 0.27 (75%) v = 1769	b = 1.546 (43%) o = 1.25 (82%) v = 2465	b = 2.247 (32%) o = 1.020 (47%) v = 4795	b = 3.066 (41%) o = 1.83 (61%) v = 5384	b = 4.154 (53%) o = 2.89 (69%) v = 5505
*Blade operating depth 75 mm*						
	b = 1.010 (16%) o = 0.81 (76%) v = 4281	b = 0.799 (19%) o = 0.52 (65%) v = 2977	b = 1.852 (41%) o = 1.31 (72%) v = 3114	b = 3.332 (32%) o = 2.24 (65%) v = 7224	b = 3.383 (41%) o = 2.32 (69%) v = 5787	b = 5.027 (47%) o = 3.18 (63%) v = 7565
*Blade operating depth 100 mm*						
	b = 1.306 (16%) o = 0.97 (74%) v = 5463	b = 1.379 (23%) o = 0.91 (67%) v = 4177	b = 3.660 (48%) o = 2.82 (77%) v = 5349	b = 50.94 (35%) o = 3.18 (64%) v = 10156	b = 5.749 (43%) o = 3.83 (64%) v = 9271 cm^3^ m^−1^	b = 8.578 (60%) o = 6.15 (72%) v = 10043

1 b = Furrow backfill expressed as the amounts of soil retained in the furrow (kg m^−1^) after strip-tillage (also expressed as %). A high backfill is desired to adequately cover seeds to ensure seed germination and reduce the risk of bird damage.

2 o = Optimum clods measured as the amount of 1–20 mm clods (kg m^−1^) retained in the furrow soil (also expressed as %). A high amount or percentage of optimum clods is desired for optimum seed-soil contact and uniform seed germination.

3 v = Volume of soil disturbance or furrow volume (cm^3^ m^−1^) measured by sand replacement method. Unnecessary soil disturbance should be avoided as it indicates energy wastage and increases soil erosion risk.

### 3.5 Soil cutting, throwing, and furrow backfilling processes

**Fig. 8:**
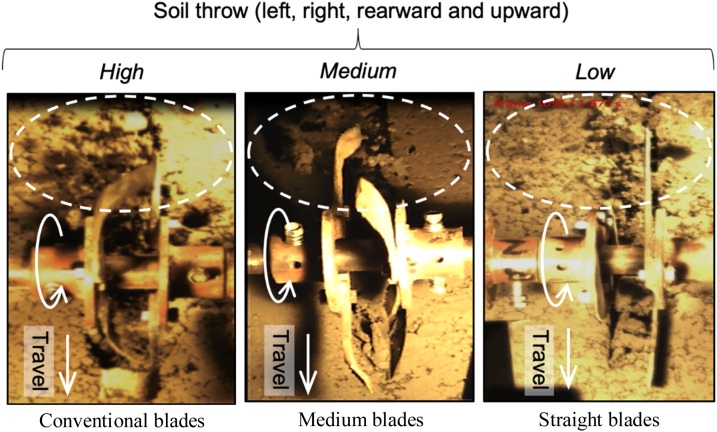
High, medium and low levels of soil throw during soil cutting by different blades as observed with high speed image acquisition. Cutting depth and blade operating depth both were 50 mm (4 blades per row).

Videography showed the sideward (left and right to the direction of travel), rearward, and upward movement of soil when strip-tilled by the three blade types (with 4 blades per row and cutting width 50 mm) in the soil bin. There was almost no soil thrown out of the tilled furrow for any of the blade types from penetration of the blades when they first entered the soil in the direction of travel. Soil breakage and throw out of the furrow that resulted from further penetration was however more considerable and showed an opposite trend. Conventional blades produced numerous shear cracks (as observed by Thakur and Godwin, 1991) during penetration and entry (Fig. 8), resulting in the production of many small clods. Conversely, the medium and straight blades generated comparatively fewer cracks and thus produced only a limited number of clods that were generally larger. It was also evident from the high speed video images that with the straight blades, the cut soil chunks were thrown mainly backward and mostly remained in the path of the successive blades for a comparatively longer period of time, thereby allowing the succeeding blades to re-cut the soil chunks repeatedly (similar to that reported by Matin et al., 2015; Saimbhi et al., 2004) and break them further into finer clods (while also throwing out some). With other two blades, the cut soil chunks were pushed strongly backward and thus they were quickly thrown out of the path of the successive blades (while also throwing out considerably) limiting soil re-cut and any further breakage.

The intensity and pattern of soil throw varied as a function of blade type during the exit phase. Conventional blades, which have the largest width and bend, threw the greatest amounts of tilled soil sideward, rearward, and upward during their exit from the furrow similar to that reported by Kataoka and Shibusawa (2002); Matin et al. (2014). The sideward and upward thrown soil clods were deposited outside of the furrow where they were unavailable as backfill, thereby decreasing seedbed quality. Conversely, most of the soil clods that were thrown backwards remained within the furrow and contributed to backfill. Overall, however, the amount of soil throw observed with conventional blades indicates that they are poorly suitable for achieving high backfill during strip-tillage, as most of the soil clods were thrown out of the furrow and these results were confirmed by observation presented in Table 1. Conversely, the straight blades, which had the least width, threw the soil clods mainly backwards where they tended to be redeposited largely within the furrow after re-cutting and breaking by the successive blades. Little sideward deposition was observed. In combination, this resulted in a comparatively higher amount of backfill (Fig. 8). The observations of the processes of soil cutting, throwing and backfilling are in consistent with findings of other studies on sandy loam and clay loam soils (Matin et al., 2015, 2016; Yang et al., 2018), though these studies were not conducted under such high soil moisture conditions. The soil cutting action of the medium blade was similar to the conventional blade, although backfill was not as great as with the straight blade, rendering it less suitable for achieving sufficient backfill. As such, the soil cutting action of the straight blade appears to be more suitable where farmers aim to attain high backfill for optimum seed coverage, which is likely to contribute to improved crop emergence and stand establishment from strip-tillage under wet clay soil conditions (Aikins et al., 2018). The use of straight blades for strip-tillage has also been recommended to achieve a high-quality furrow seed bed (Matin et al., 2014, 2016) or reducing energy requirement (Matin et al., 2015).

### 3.6 Implications

The commercially available blade designs found in Asian 2WT machinery markets are mainly C-shaped and L-shaped, though others such as I-type, scoop-type and straight-type are also available (Asl and Singh, 2009; Beeny and Khoo, 1970; Lee et al., 2003; Matin et al., 2015, 2016). There is a wide variation in design dimensions of these blades and their performance varies depending on soil type and soil moisture conditions (Aikins et al., 2018; Matin et al., 2014, 2016). Lee et al. (2003) reported that the rotary blade with four blades per row had highest soil breakage (24.4%), optimum width of soil cutting (80 mm) and required lowest torque, and that 4 blades per row was optimum for direct seeding on a sandy loam in Korea. Asl and Singh (2007a, 2007b) concluded that power consumption increased from two to four blades per row and that the latter was optimal for strip-tillage. Ahmad (1986) also concluded that with increase in cutting width (i.e., more blades per row) beyond 50 mm, there was a significant increase in power consumption, but reduction of specific power requirement. He described several relationships among forward and rotor speeds, bite length and cutting width of the blades that would determine the power consumption and energy requirements for strip-tillage, concluded that smaller cutting widths, smaller bite lengths and higher rotor speeds would increase the degree of soil breakage, though smaller cutting widths and lower rotor speeds would be required to lower energy requirements in sandy clay loams.

The shape of the rotary blade also affects the quality of soil tilth and the energy requirements of strip-tillage. Chertkiattipol et al. (2008) showed no significant differences in soil clod diameter between the three rotary tillers (i.e., Japanese C-shaped and two new prototype rotary tillers) but the soil clod diameter decreased, and soil inversion increased with increasing rotational speed of the rotor. Chertkiattipol and Niyampa (2010) reported that the shape of the rotary blade influenced torque characteristics and specific tilling energy, with the latter higher for the Japanese C-shaped than the European L-shaped blade on sandy clay loam and clay soils in Thailand.

Our findings indicate that both conventional and straight blades were efficient in cutting wet soil and producing favorable U-shaped strip-till furrows with a high percentage of optimum soil clods (1–20 mm size) for seeding. Straight blades however had higher amount of backfill required to cover seeds in furrows. Increasing the operating depth of all the blades increased the furrow depth, backfill and optimum clods and increased furrow uniformity. Increasing the cutting width increased the amounts of optimum clods (as more blades were involved in soil cutting) and reduced the unwanted fine particles significantly. Increased cutting width is associated with increased energy requirements (Ahmad, 1986; Chamen and Cope, 1979; Perdok and Burema, 1977). Such improvement in furrow seedbed parameters in clayey soils can facilitate early planting of winter season crops to replace fallows after monsoon rice, as well as in intensively cropped areas, while reducing production costs and energy inefficiency (Chertkiattipol and Niyampa, 2010; Matin et al., 2015).

## 4 Conclusions and recommendation

Increasing the cropping intensity by growing winter or *rabi* crops in place of fallows after monsoon rice through timely and mechanized planting tends to be challenged in South Asia by wet and heavy soil conditions. As traditional farm machinery delays planting, the current study aimed to improve the rotary strip-till blade design and setting, and tested three blade designs (conventional or bent C, medium or half-width bent C, and straight or straight C) at three blade operating depths (50, 75 and 100 mm) and two blade settings or cutting widths (4 or 6 blades per row; 50 and 100 mm) on a sandy clay loam soil in southern Bangladesh. Our results suggest that among the three blades tested, straight blades have an improved ability to till a deeper furrow and capture a high amount of backfill and optimum-sized soil clods compared to other blades, indicative of their suitability for four straight blades per row rotavator configurations. These specifications are likely to reduce fuel consumption and enhance emergence and stand establishment under strip-tillage on such soils. Further work is however recommended to confirm these results under field conditions including crop residue retention. Slightly longer blades may also be needed to provide a rotor diameter of 420–450 mm to till deeper at a depth between 75 and 100 mm so the rotor shaft or blade holders do not touch the ground or rake residues. These results have implications for future design of improved machinery and their evaluation for strip-tillage and cropping systems intensification in rice-based rotational systems in coastal South Asia. Efforts to increase manufacturing and commercial availability of straight blade designs for 2WT seed drills are now needed to aid in achieving the formation of improved tilth, increased furrow backfill, and improved seed-soil contact under on-farm conditions.

## Disclaimer

The authors indicate no conflict of interest.

## Declaration of Competing Interest

The authors report no declarations of interest.
